# Effect of Acupuncture on Heart Rate Variability: A Systematic Review

**DOI:** 10.1155/2014/819871

**Published:** 2014-02-12

**Authors:** Joanne W. Y. Chung, Vincent C. M. Yan, Hongwei Zhang

**Affiliations:** ^1^Department of Health and Physical Education, The Hong Kong Institute of Education, 10 Lo Ping Road, Tai Po, New Territories, Hong Kong; ^2^Chung Chi College, Chinese University of Hong Kong, Room 540, Sino Building, Shatin, Hong Kong

## Abstract

*Aim.* To summarize all relevant trials and critically evaluate the effect of acupuncture on heart rate variability (HRV). *Method.* This was a systematic review with meta-analysis. Keyword search was conducted in 7 databases for randomized controlled trials (RCTs). Data extraction and risk of bias were done. *Results.* Fourteen included studies showed a decreasing effect of acupuncture on low frequency (LF) and low frequency to high frequency ratio (LF/HF ratio) of HRV for nonhealthy subjects and on normalized low frequency (LF norm) for healthy subjects. The overall effect was in favour of the sham/control group for high frequency (HF) in nonhealthy subjects and for normalized high frequency (HF norm) in healthy subjects. Significant decreasing effect on HF and LF/HF ratio of HRV when acupuncture was performed on ST36 among healthy subjects and PC6 among both healthy and nonhealthy subjects, respectively. *Discussion.* This study partially supports the possible effect of acupuncture in modulating the LF of HRV in both healthy and nonhealthy subjects, while previous review reported that acupuncture did not have any convincing effect on HRV in healthy subjects. More published work is needed in this area to determine if HRV can be an indicator of the therapeutic effect of acupuncture.

## 1. Introduction

Acupuncture has been described as a philosophy-based, not science-based, medicine [1]. Probably, this is because the philosophy underpinning Chinese medicine reflects heavily oriental culture and philosophy. It emphasizes a holistic view embracing both internal and external environment of an individual. Acupuncture is a commonly practiced Chinese medicine therapeutic modality. The mechanism of acupuncture is the restoration of a harmonious flow of *qi* (life energy) by manipulating the complementary and opposing elements of yin and yang so as to maintain its balance throughout the body. The balance and harmonious flow of *qi *are believed to be the basis of good health [2].

The Western medical community now accepts that acupuncture works, at least for the relief of pain, but it still struggles to translate the mechanism of acupuncture into the Western paradigm. In recent decades, the increasing popularity of Chinese medicine in general and acupuncture in particular among the public around the world has motivated the scientific community to undertake intensive investigation of acupuncture's efficacy, as well as the physiological basis behind it. After decades if not centuries of treating individual problems, the Western medical community is coming to understand that human health is a holistic phenomenon reflecting balance—or imbalance—in the bio-psycho-social-spiritual aspects of life. While this balance reflects complex factors, there are parameters we can measure—there are physical indications—of how an individual responds to a disease state in order to maintain the bio-psycho-social-spiritual balance within our body. Heart rate variability (HRV) is a measure of this kind.

**Figure 1:**
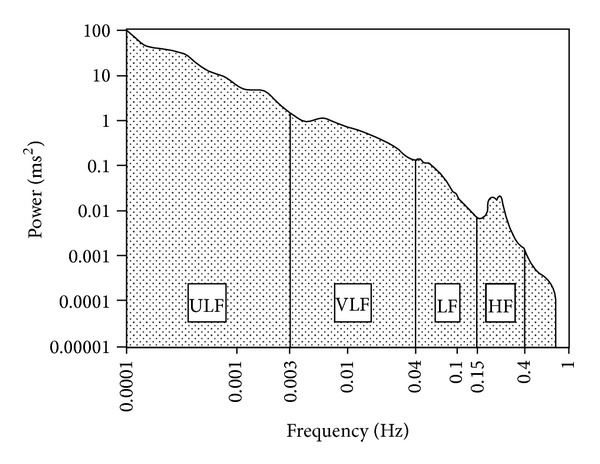
Diagram of the power spectral density heart rate variability (HRV) (adapted from [4]).

HRV can be measured by time domain analysis or frequency domain analysis as a power spectrum produced by a selected chain of heart to heart beat. Using power spectral analysis [3], Figure 1 shows an estimate of power spectral density obtained from the entire 24-hour interval of a long-term Holter recording. Only the low frequency (LF) and high frequency (HF) components correspond to peaks of the spectrum, while the very low frequency (VLF) and ultra-low frequency (ULF) can be approximated by a line in this plot with logarithmic scales on both axes. The slope of such a line is the *α* measure of HRV [4].

According to [4], VLF, LF, HF, and LF/HF ratios are the common parameters used in assessing the autonomic system (Table 1).

HRV measures the balance of our autonomic nervous system which reflects physiological, hormonal, and emotional balance. The autonomic nervous system is composed of two branches, namely, sympathetic nervous system and parasympathetic nervous system. Sympathetic nervous system is responsible for flight and stress situation while parasympathetic nervous system is dominant when relaxed [5]. Hence, HRV measures the balance of the activities of the sympathetic nervous system and the parasympathetic nervous system. Researchers have hypothesized that acupuncture modulates the autonomic nervous system, thereby revitalizing the balance of metabolism [6–9]. These modulations and revitalizing effects can be measured by HRV.

The autonomic nervous system dynamically controls the response of the body to a range of external and internal stimuli, providing physiological stability [10]. HRV is a measure of the naturally occurring beat-to-beat changes in heart rate [11]. It serves as a critical method for gauging human health and resiliency. HRV measures regulation of the heart by the autonomic nervous system [9]. In other words, HRV analysis reveals the interaction between the sympathetic and parasympathetic activities by modulation of heart beat interval [7].

HRV is an established tool in cardiology research and is being increasingly used for a range of clinical applications [10]. For example, HRV decreases in congestive heart failure [12]. HRV power spectral analysis shows that the LF component diminishes in patients with congestive heart failure, and the decrease is related to an increased risk of sudden death [12]. This was supported by [12] in which they reported that dogs with a reduction in LF power and LF/HF ratio observed in heart failure dogs had diminished sinus node responsiveness to autonomic modulation.

Both timedomain heart rate variability and respiration rate in various positions—for example, lying down in a laboratory, cycling in a laboratory, and sleeping in an ambulatory surrounding—showed high intraclass correlation coefficients [13]. Therefore, HRV is a reliable tool for tracking changes of clinical state.

Acupuncture is generally documented to be effective in treating chronic and acute pain conditions by inserting needles into specific “acupuncture points” (acupoints) on the patient's body [14]. Acupuncture analgesia can be described in technical terms as a manifestation of integrative processes at different levels in the central nervous system between afferent impulses from pain regions and impulses from acupoints. Recent research has suggested different mechanisms for acupuncture. For instance, HRV results from the regulation of the heart by the autonomic nervous system. However, the interrelationship between these mechanisms and how acupuncture affects complex physiological systems is still not understood [9].

Previous studies have showed a relationship between acupuncture and heart rate variability. Wang et al. reported that acupuncture enhances cardiac vagal activity and suppresses sympathetic activities in healthy humans [15]. In other words, the LF decreased. This is consistent with another study also conducted with healthy adults. The study reported that acupuncture significantly increased HF and decreased both the LF/HF ratio and heart rate [16]. The result supports the hypothesis that acupuncture affects parasympathetic activity.

Several studies reported the effect of acupuncture on HRV using specified acupoints. For instance, acupuncture on Neiguan (PC6) and Gongsun (SP4) decreased sympathetic activity and balanced the autonomic nervous system for those who were under stress [17]. Similarly, acupuncture on Neiguan (PC6) and Shenmen (HT7) among patients with poststroke onset insomnia was reported to be effective in that there was a reduction in sympathetic hyperactivities after the acupuncture [18]. A study comparing different acupuncture methods (needle, laser acupuncture, moxibustion, and teleacupuncture) on 72 patients to evaluate the influence of acupuncture on heart rhythm over the short-term and long-term measurements [19]. Significant decreases in heart rate after *verum* intervention at Yintang, Neiguan, and Guanyuan were found. Improvements in state of health following teleacupuncture were shown. HRV analysis was demonstrated to be effective in evaluating the status of health during acupuncture [19]. However, Wright and Aickin reported essentially no support for a relationship between HRV and acupuncture intervention [20]. In another study with 16 acupoints, acupuncture intervention did not show any generalized depressor effect on the autonomic nervous system [21]. To date, only one systematic review evaluated the effect of acupuncture on HRV [8]. Results, yet, were contradictory.

To understand the mechanism of acupuncture, we need to understand how clinical scientific studies were done and how acupuncture established its effects for some specific health conditions. A well-designed trial, therefore, isolates the specific treatment variable to see what effect it has. To this end, a systematic review investigating (a) the effect of acupuncture on HRV and (b) the potential for using HRV as an indicator of the therapeutic effect of acupuncture was conducted in this study.

## 2. Methods and Materials

### 2.1. Study Design

It was a systematic review with meta-analysis.

### 2.2. Inclusion and Exclusion Criteria

In this research, randomized control trials (RCTs) were included.  Table 2 shows the inclusion criteria of the RCTs that were selected.

There was no language restriction and only papers with fulltext were included for analysis. Studies were excluded if trials testing forms of acupuncture other than needle acupuncture, for example, laser acupuncture or moxibustion; and, if there was only one acupuncture session reported in the studies.

### 2.3. Search Strategy: Keywords and Database

The following keywords were used in the database search: acupuncture OR meridian OR acupoint AND heart rate variability or HRV or heart rate. For Chinese databases, keywords in Chinese used were* acupuncture OR meridian OR acupoint OR electroacupuncture AND Heart rate OR heart rate variability.*

Databases searched were CINAHL (1937-), EMbase (1980-), PsycInfo (1597-), Cochrane Central Register of Controlled Trials (1898-), Ovid Medline (1950-), Korean Studies Info Services System, and China Academic Journals Fulltext Database (CAJ) (1915-).

### 2.4. Study Identification

The initial search yielded 94, 848, 30, 155, 89, 3, and 1 paper from CINAHL, EMbase, PsycInfo, Cochrane Central Register of Controlled Trials, Ovid Medline, Korean Studies Info Services System, and CAJ, respectively. Then, the researcher screened the title and the abstract to determine the relevancy. As a result, 1,220 articles were found and comprised the initial data extraction.

### 2.5. Data Extraction

The researcher designed a data extraction form, and the two independent reviewers completed the data extraction according to the topics predetermined in the form (Table 3). These topics were (1) first author and year, (2) design, sample size, conditions, (3) intervention, (4) heart rate variability, (5) main results and intergroup differences, (6) respiratory regulation, measuring position, measuring time, and (7) risk of bias [8].

### 2.6. Assessment of the Risk of Bias

Cochrane criteria were used to assess the risk of bias of the studies [34]. The assessment comprised evaluation of randomization, blinding, withdrawals, and blinding and allocation concealment. As pointed out by [8], it is technically difficult to blind the therapist in the application of acupuncture; therefore, the researcher divided “blinded” into “blinded patients” (which is possible) and “blinded therapist” (which is nearly impossible).

Hard copies of all the articles with full text were printed and studied by two independent reviewers. The two independent reviewers agreed on the assessment of risk of bias. The results of the two reviewers were the same, and the results are shown in Table 3.

### 2.7. Data Synthesis

Chi-squared test and Higgins *I* ^2^ tests were used to evaluate heterogeneity. To compare the mean changes in the HRV parameters, the researcher computed the weighted mean differences (WMDs) for outcome on the same scale and standardized mean differences (SMDs) for outcomes on a different scale. A summary of estimates of the treatment effect was calculated using the more conservative approach of a random effects model. Forest plots were done to display the treatment effect visually. The analysis was conducted using Revman 5.0 software.

## 3. Results

### 3.1. Study Description

**Figure 2:**
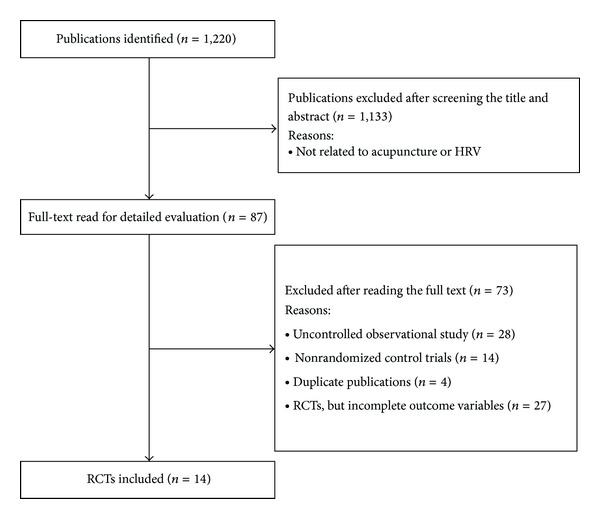
Process of studies selection.

Using the keyword search, the researcher identified 1220 articles initially. Upon reading the abstracts, 1133 articles were excluded because they were not related to acupuncture or HRV. The remaining 87 articles in full text were then reviewed by the two reviewers. Seventy-three articles were excluded because of non-RCTs and incomplete outcome measures. Fourteen studies were included in the analysis (Figure 2). Among the 14 studies, 2 were from South Korea, 6 from Taiwan, 2 from USA, 1 from Germany, 2 from China, and 1 from Japan. Slightly less than a quarter of them (10/14 = 71.42%) were of crossover design, and 4 were of parallel design. Eleven studies involved healthy subjects, 2 involved patients with functional dyspepsia, and 1 involved patients with coronary heart disease. All studies used LF, HF, and LF/HF as their outcome measures; some had LF norm and HF norm. Therefore, these measures were used in the meta-analysis as long as data were sufficient for computation.

### 3.2. Risk of Bias

**Figure 3:**
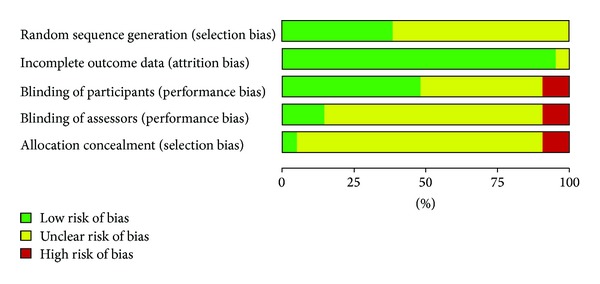
Results of risk of bias analysis.

For risk of bias evaluation, the researcher used the following criteria: sequence generation, incomplete data, patient-blinded, assessor-blinded, and allocation concealment (Table 3). These criteria were evaluated and rated as unknown (U), criteria met (Y), and criteria not met (N). For sequence generation, 11 (78.57%) studies rated U and 3 rated Y (21.43%). For incomplete data, 11 studies rated U (78.57%) and 3 rated Y (21.43%). For patient-blinded, 11 studies rated U (78.57%) and 3 rated Y (21.43%). For assessor blinded, 11 studies rated U (78.57%), 1 rated N (7.14%), and 2 rated Y (14.29%). For allocation concealment, 3 studies (21.43%) rated U, 1 rated N (7.14%) and 10 rated Y (71.49%). None of the studies scored Y for all criteria while 1 study got all U.  Figure 3 shows the results of risk of bias analysis. The studies without patient-blinded, assessor-blinded or allocation concealment were subjected to high risk of bias.

### 3.3. Outcome Measures

Fourteen studies meeting the selection criteria were included in the study. It is noted that some of the included studies had more than one group, that is, acupuncture, sham, and control. In view of the fact that all the tests in the groups had the same outcome measures, the researcher entered the outcome measures of the respective group as one study ID in the Revman 5.0. Six outcome measures were input for analysis, namely, HF, LF and LF/HF ratio, HF norm, and LF norm. Thus, there may be more than one study ID with the same author and year entry in the subsequent meta-analysis for one included study.

### 3.4. Healthy Subject Studies

#### 3.4.1. High Frequency (7 Studies)

**Figure 4:**
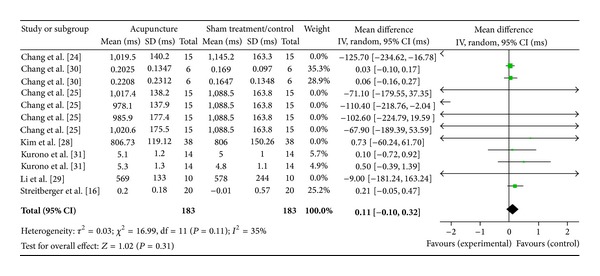
Forest plot of the effects of acupuncture on HF for healthy subjects.

Seven studies were included in this part of the analysis, corresponding to 16 study IDs, as explained in the previous section (Figure 4). There were 183 subjects in acupuncture group and 183 in sham/control group. The heterogeneity (*χ* ^2^ = 16.99, df = 11, *P* = 0.11) was not significant while the *I* ^2^ was 35% which means low heterogeneity. The overall effect was insignificant (*Z* = 1.02, *P* = 0.31). All the 95% CI of the WMDs of the studies and the overall effect (diamond) crossed the line of no effect.

#### 3.4.2. Low Frequency (6 Studies)

**Figure 5:**
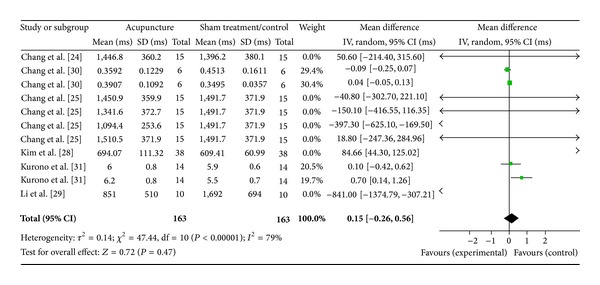
Forest plot of the effects of acupuncture on LF for healthy subjects.

Six studies were evaluated in this section of the study, while there were 13 study IDs in this analysis (Figure 5). There were 163 subjects in acupuncture group and 163 in sham/control group. The heterogeneity (*χ* ^2^ = 47.44, df = 10, *P* < 0.00) was significant while the *I* ^2^ was 79% which means high heterogeneity. Though the heterogeneity was high, the overall effect of the acupuncture in the included studies was insignificant (*Z* = 0.72, *P* = 0.47). All the 95% CI of the weighted mean difference of the studies and the overall effect (diamond) crossed with line of no effect.

#### 3.4.3. LF/HF Ratio (9 Studies)

**Figure 6:**
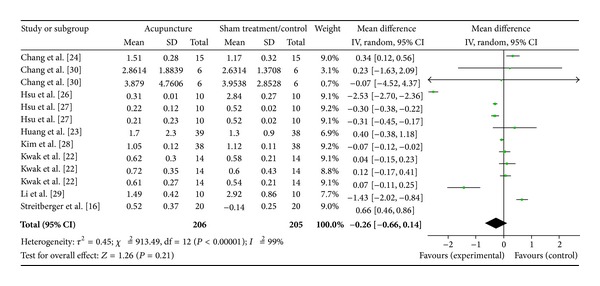
Forest plot of the effects of acupuncture on LF/HF ratio for healthy subjects.

Nine studies were evaluated in this section of the study; for these 9 studies, there were 13 study IDs (Figure 6). There were a total of 206 subjects in the acupuncture groups of these studies and a total of 205 in the sham/control groups. The heterogeneity (*χ* ^2^ = 913.49, df = 12, *P* < 0.00) was significant while the *I* ^2^ was 99% which was high heterogeneity. The overall effect was not significant (*Z* = 1.26, *P* = 0.21). Results in Chang et al. [24] and Streitberger et al. [16] did not support that effect of acupuncture while Hsu et al. [27], Hsu et al. [27], Kim et al. [28], and Li et al. [29] did. However, the overall effect (diamond) was insignificant and crossed the line of no effect.

#### 3.4.4. HF Norm (5 Studies)

**Figure 7:**
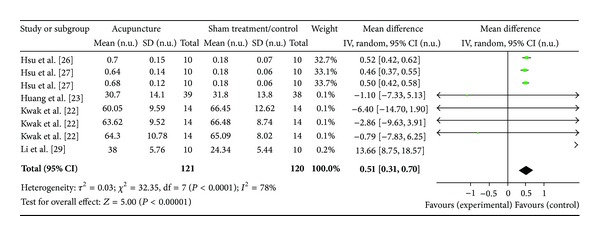
Forest plot of the effects of acupuncture on HF norm for healthy subjects.

Five studies were evaluated in this section of the study, corresponding to 8 study IDs (Figure 7). There were 121 subjects in acupuncture group and 120 in sham/control group. The heterogeneity (*χ* ^2^ = 32.35, df = 7, *P* < 0.00) was significant while the *I* ^2^ was 78% which means high heterogeneity. The overall effect was significant (*Z* = 5.00, *P* < 0.00). Hsu et al. [26], Hsu et al. [27], Hsu et al. [27] did not support the effect of acupuncture on HF norm, and the overall effect was significant and was in favour of the sham/control group with decreased HF norm as shown in the WMDs.

#### 3.4.5. LF Norm (5 Studies)

**Figure 8:**
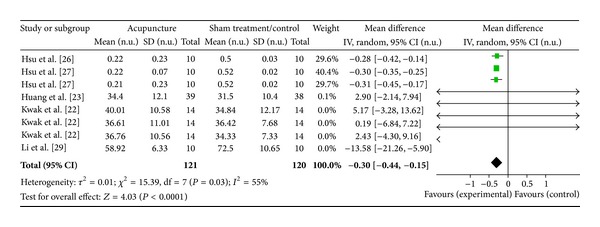
Forest plot of the effects of acupuncture on LF norm for healthy subjects.

Five studies were included while there were 8 study IDs in this analysis (Figure 8). There were 121 subjects in acupuncture group and 120 in sham/control group. The heterogeneity (*χ* ^2^ = 15.39, df = 7, *P* = 0.03) was significant while the *I* ^2^ was 55% which means moderate heterogeneity. The overall effect was significant (*Z* = 4.03, *P* < 0.00). Results showed a decreased effect of acupuncture on LF norm [26, 27] while the rest showed no effect. The overall effect (diamond) supported the conclusion that acupuncture had a significant effect on LF norm with a decreased magnitude in the WMDs.

#### 3.4.6. ST36 on HF (2 Studies)

**Figure 9:**
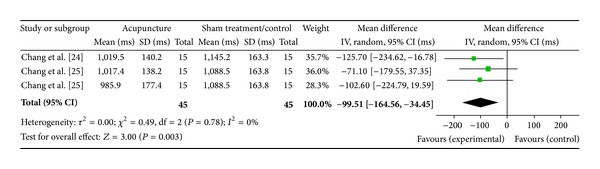
Forest plot of the effects of acupuncture at ST36 on HF for healthy subjects.

Two studies were included while there were 3 study IDs in this analysis (Figure 9). There were 45 subjects in acupuncture group and 45 in sham/control group. The heterogeneity (*χ* ^2^ = 0.49, df = 2, *P* = 0.78) was not significant while the *I* ^2^ was 0% which means no heterogeneity. The overall effect was significant (*Z* = 3.00, *P* = 0.003). Results showed a decreased effect of acupuncture at ST36 on HF while the rest showed no effect. The overall effect (diamond) supported significant effect acupuncture of ST36 on HF and showed a decreased magnitude in the WMDs.

### 3.5. Nonhealthy Subject Studies

#### 3.5.1. High Frequency (2 Studies)

**Figure 10:**
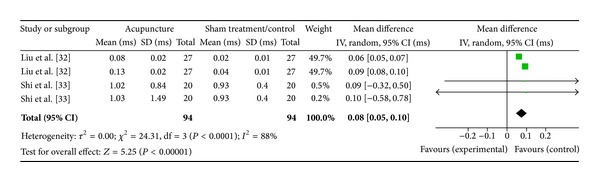
Forest plot of the effects of acupuncture on HF for nonhealthy subjects.

Two studies were included while there were 4 study IDs in this analysis (Figure 10). There were 94 subjects in acupuncture group and 94 in sham/control group. The heterogeneity (*χ* ^2^ = 24.31, df = 3, *P* < 0.00) was significant while the *I* ^2^ was 88% which means high heterogeneity. The overall effect was significant (*Z* = 5.25, *P* < 0.00). Results of the WMDs showed a significant decrease of HF in the sham/control group [32, 33]. The overall effect (diamond) favoured the control; thus, effect of acupuncture on HF was not supported.

#### 3.5.2. Low Frequency (1 Study)

**Figure 11:**
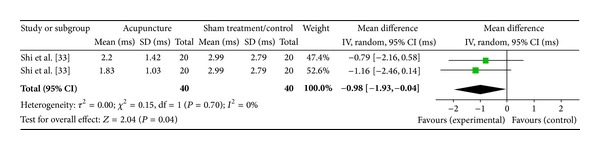
Forest plot of the effects of acupuncture on LF for nonhealthy subjects.

One study was included while there were 2 study IDs in this analysis (Figure 11). There were 40 subjects in acupuncture group and 40 in sham/control group. The heterogeneity (*χ* ^2^ = 0.15, df = 1, *P* = 0.70) was not significant while the *I* ^2^ was 0% because there was only one study. The overall effect was significant (*Z* = 2.04, *P* = 0.04). The results of the WMDs [33] showed effect of a decreased LF. The overall effect (diamond) was marginally significant.

#### 3.5.3. LF/HF Ratio (2 Studies)

**Figure 12:**
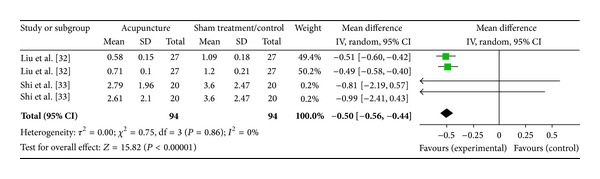
Forest plot of the effects of acupuncture on LF/HF ratio for nonhealthy subjects.

Two studies were included while there were 4 study IDs in this analysis (Figure 12). There were 94 subjects in acupuncture group and 94 in sham/control group. The heterogeneity (*χ* ^2^ = 0.75, df = 3, *P* = 0.86) was not significant while the *I* ^2^ was 0%. The overall effect was significant (*Z* = 15.82, *P* < 0.00). Results of the WMDs and the overall effect (diamond) showed a significant effect of acupuncture on LF/HF ratio as in Liu et al. [32]. The LF/HF ratio was decreased.

#### 3.5.4. PC6 on HF (2 Studies)

**Figure 13:**
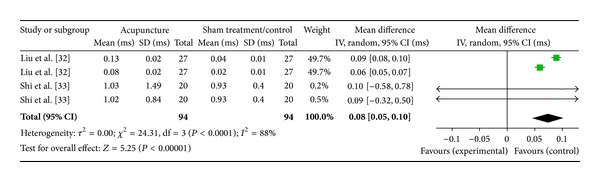
Forest plot of the effects of acupuncture at PC6 on HF for nonhealthy subjects.

Two studies were included while there were 4 study IDs in this analysis (Figure 13). There were 94 subjects in acupuncture group and 94 in sham/control group. The heterogeneity (*χ* ^2^ = 24.31, df = 3, *P* < 0.00) was significant while the *I* ^2^ was 88%. It showed high heterogeneity. The overall effect was significant (*Z* = 5.25, *P* < 0.00). With reference to WMDs, the significant effect of sham at PC6 on decreased HF was reported, not on the acupuncture group.

#### 3.5.5. PC6 on LF/HF (2 Studies)

**Figure 14:**
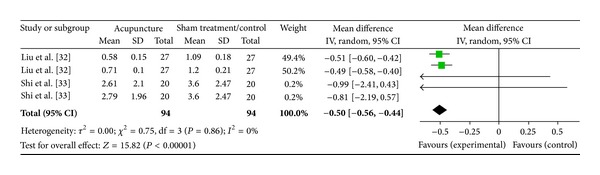
Forest plot of the effects of acupuncture at PC6 on LF/HF for nonhealthy subjects.

Two studies were included while there were 4 study IDs in this analysis (Figure 14). There were 94 subjects in acupuncture group and 94 in sham/control group. The heterogeneity (*χ* ^2^ = 0.75, df = 3, *P* = 0.86) was not significant while the *I* ^2^ was 0%. It showed no heterogeneity. The overall effect was significant (*Z* = 15.83, *P* < 0.00). With reference to WMDs, the significant effect of acupuncture at PC6 on decreased LF/HF was reported.

### 3.6. Combined Analysis for Healthy and Nonhealthy Subjects Studies

#### 3.6.1. LI4 on High Frequency (2 Studies)

**Figure 15:**
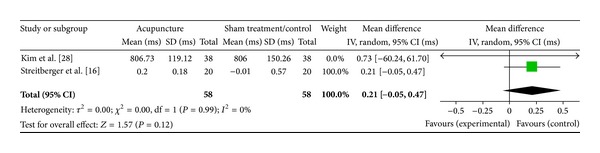
Forest plot of the effects of acupuncture at LI4 on HF for all healthy and nonhealthy subjects.

Two studies were included while there were 2 study IDs in this analysis (Figure 15). There were 58 subjects in acupuncture group and 58 in sham/control group. The heterogeneity (*χ* ^2^ = 0.00, df = 1, *P* = 0.99) was not significant while the *I* ^2^ was 0%. It showed no heterogeneity. The overall effect was significant (*Z* = 1.57, *P* = 0.12) on sham/control only which was contributed by Kim et al. [28] and Streitberger et al. [16]. Results did not support the effect of acupuncture at LI4 on decreased HF.

#### 3.6.2. LI4 on LF/HF Ratio (4 Studies)

**Figure 16:**
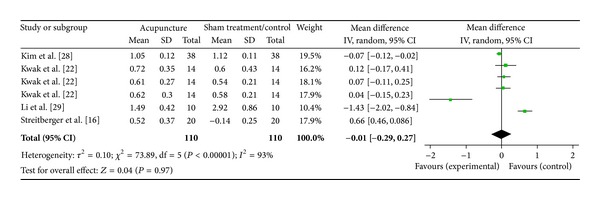
Forest plot of the effects of acupuncture at LI4 on LF/HF ratio for all healthy and nonhealthy subjects.

Four studies were included while there were 6 study IDs in this analysis (Figure 16). There were 110 subjects in acupuncture group and 110 in sham/control group. The heterogeneity (*χ* ^2^ = 73.89, df = 5, *P* < 0.00) was significant while the *I* ^2^ was 93%. It showed high heterogeneity. The overall effect was not significant (*Z* = 0.04, *P* = 0.97). Results did not support the effect of acupuncture at LI4 on decreased LF/HF ratio.

#### 3.6.3. LI4 on HF Norm (2 Studies)

**Figure 17:**
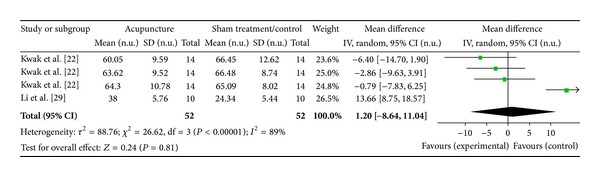
Forest plot of the effects of acupuncture at LI4 on HF norm for all healthy and nonhealthy subjects.

Two studies were included while there were 4 study IDs in this analysis (Figure 17). There were 52 subjects in acupuncture group and 52 in sham/control group. The heterogeneity (*χ* ^2^ = 26.62, df = 3, *P* < 0.00) was significant while the *I* ^2^ was 89%. It showed high heterogeneity. The overall effect was not significant (*Z* = 0.24, *P* = 0.81). Results did not support the effect of acupuncture at LI4 on decreased HF norm.

#### 3.6.4. LI4 on LF Norm (2 Studies)

**Figure 18:**
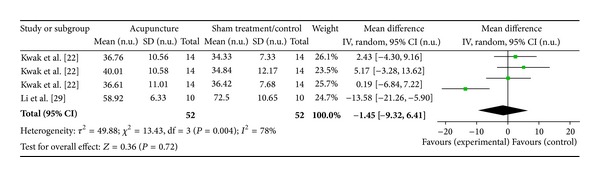
Forest plot of the effects of acupuncture at LI4 on LF norm for all healthy and nonhealthy subjects.

Two studies were included while there were 4 study IDs in this analysis (Figure 18). There were 52 subjects in acupuncture group and 52 in sham/control group. The heterogeneity (*χ* ^2^ = 13.43, df = 3, *P* = 0.004) was significant while the *I* ^2^ was 78%. It showed moderate heterogeneity. The overall effect was not significant (*Z* = 0.36, *P* = 0.72). Results did not support the effect of acupuncture at LI4 on decreased LF norm.

#### 3.6.5. PC6 on HF (3 Studies)

**Figure 19:**
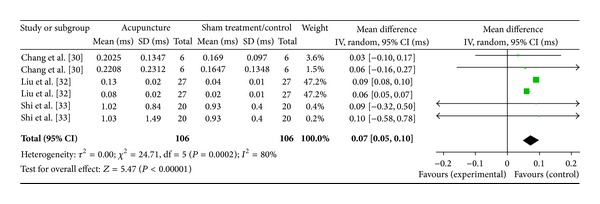
Forest plot of the effects of acupuncture at PC6 on HF for all healthy and nonhealthy subjects.

Three studies were included while there were 6 study IDs in this analysis (Figure 19). There were 106 subjects in acupuncture group and 106 in sham/control group. The heterogeneity (*χ* ^2^ = 24.71, df = 5, *P* = 0.0002) was significant while the *I* ^2^ was 80%. It showed high heterogeneity. The overall effect was significant (*Z* = 5.47, *P* < 0.00). With reference to WMDs, the significant effect of acupuncture at PC6 on sham decreased HF was contributed by Chang et al. [30], Liu et al. [32], and Shi et al. [33].

#### 3.6.6. PC6 on LF (2 Studies)

**Figure 20:**
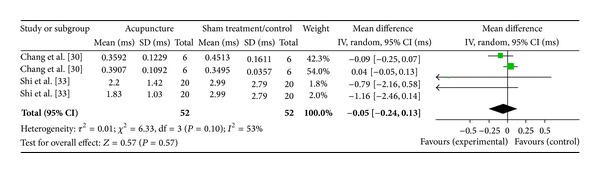
Forest plot of the effects of acupuncture at PC6 on LF for all healthy and nonhealthy subjects.

Two studies were included while there were 4 study IDs in this analysis (Figure 20). There were 52 subjects in acupuncture group and 52 in sham/control group. The heterogeneity (*χ* ^2^ = 6.33, df = 3, *P* = 0.10) was not significant while the *I* ^2^ was 53%. It showed low heterogeneity. The overall effect was not significant (*Z* = 0.57, *P* = 0.57). Results did not support the effect of acupuncture at PC6 on decreased LF.

#### 3.6.7. PC6 on LF/HF Ratio (6 Studies)

**Figure 21:**
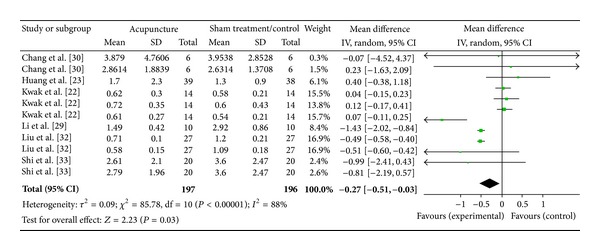
Forest plot of the effects of acupuncture at PC6 on LF/HF ratio for all healthy and nonhealthy subjects.

Six studies were included while there were 11 study IDs in this analysis (Figure 21). There were 197 subjects in acupuncture group and 196 in sham/control group. The heterogeneity (*χ* ^2^ = 85.78, df = 10, *P* < 0.00) was significant while the *I* ^2^ was 88%. It showed high heterogeneity. The overall effect was significant (*Z* = 2.23, *P* = 0.03). With reference to WMDs, the significant effect of acupuncture at PC6 on acupuncture group decreased LF/HF ratio was contributed by Chang et al. [30], Huang et al. [23], Kwak et al. [22], Liu et al. [32], and Shi et al. [33].

#### 3.6.8. PC6 on HF Norm (3 Studies)

**Figure 22:**
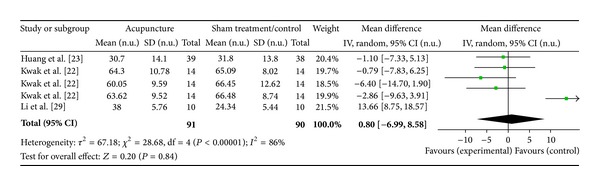
Forest plot of the effects of acupuncture at PC6 on HF norm for all healthy and nonhealthy subjects.

Three studies were included while there were 5 study IDs in this analysis (Figure 22). There were 91 subjects in acupuncture group and 90 in sham/control group. The heterogeneity (*χ* ^2^ = 67.18, df = 4, *P* < 0.00) was significant while the *I* ^2^ was 86%. It showed high heterogeneity. The overall effect was not significant (*Z* = 0.20, *P* = 0.84). Results did not support the effect of acupuncture at PC6 on decreased HF norm.

#### 3.6.9. PC6 on LF Norm (3 Studies)

**Figure 23:**
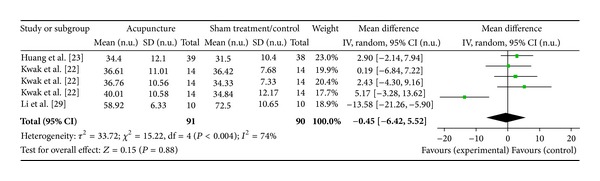
Forest plot of the effects of acupuncture at PC6 on LF norm for all healthy and nonhealthy subjects.

Three studies were included while there were 5 study IDs in this analysis (Figure 23). There were 91 subjects in acupuncture group and 90 in sham/control group. The heterogeneity (*χ* ^2^ = 15.22, df = 4, *P* = 0.004) was significant while the *I* ^2^ was 74%. It showed moderate heterogeneity. The overall effect was not significant (*Z* = 0.15, *P* = 0.88). Results did not support the effect of acupuncture at PC6 on decreased LF norm.

#### 3.6.10. ST36 on High Frequency (3 Studies)

**Figure 24:**
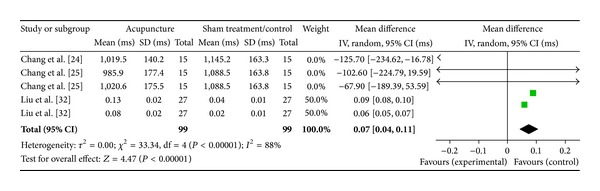
Forest plot of the effects of acupuncture at ST36 on HF for all healthy and nonhealthy subjects.

Three studies were included while there were 5 study IDs in this analysis (Figure 24). There were 99 subjects in acupuncture group and 99 in sham/control group. The heterogeneity (*χ* ^2^ = 33.34, df = 4, *P* < 0.00) was significant while the *I* ^2^ was 88%. It showed high heterogeneity. The overall effect was significant (*Z* = 4.47, *P* < 0.00). With reference to WMDs, the significant effect of acupuncture at ST36 on sham group decreased HF was contributed by Chang et al. [24], Chang et al. [25] and Liu et al. [32].

#### 3.6.11. ST36 on Low Frequency (2 Studies)

**Figure 25:**
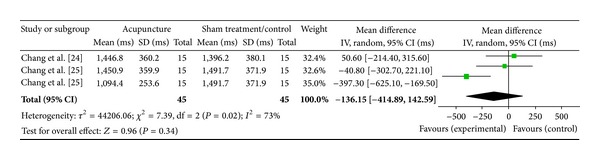
Forest plot of the effects of acupuncture at ST36 on LF for all healthy and nonhealthy subjects.

Two studies were included while there were 3 study IDs in this analysis (Figure 25). There were 45 subjects in acupuncture group and 45 in sham/control group. The heterogeneity (*χ* ^2^ = 7.39, df = 2, *P* = 0.02) was significant while the *I* ^2^ was 73%. It showed moderate heterogeneity. The overall effect was not significant (*Z* = 0.96, *P* = 0.34). Results did not support the effect of acupuncture at ST36 on decreased LF.

#### 3.6.12. ST36 on LF/HF Ratio (3 Studies)

**Figure 26:**
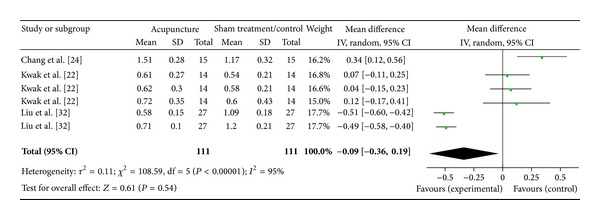
Forest plot of the effects of acupuncture at ST36 on LF/HF ratio for all healthy and non-healthy subjects.

Three studies were included while there were 6 study IDs in this analysis (Figure 26). There were 111 subjects in acupuncture group and 111 in sham/control group. The heterogeneity (*χ* ^2^ = 108.59, df = 5, *P* < 0.00) was significant while the *I* ^2^ was 95%. It showed high heterogeneity. The overall effect was not significant (*Z* = 0.61, *P* = 0.54). Results did not support the effect of acupuncture at ST36 on decreased LF/HF ratio.

### 3.7. Summary of Effect of Acupuncture on HRV

Table 4 provides a summary of results with regard to heterogeneity of the studies. All the studies on related HRV outcome measures showed moderate to high heterogeneity. This supported the use of a random effect model in meta-analysis.  Table 5 shows the effect of acupuncture on LF and LF/HF ratio for nonhealthy subjects and its effect on LF norm for healthy subjects. Considering the WMDs, we find that the overall effect was in favour of the sham/control, not the acupuncture group, for HF in nonhealthy subjects and for HF norm in healthy subjects. For effect of HRV when acupoints were stimulated, significant decreasing effect on HF component of HRV when acupuncture was performed on ST36 among healthy subjects and on PC6 among both healthy and nonhealthy subjects. For nonhealthy subjects, studies found a decreasing effect on HF component of HRV when sham acupuncture was performed on PC6 and a decreasing effect on LF/HF component of HRV when actual acupuncture was performed on PC6.

## 4. Discussion

### 4.1. Comparison with Previous Findings

To date, there has been one published systematic review which studied the effect of acupuncture on HRV [8]. The study reported that acupuncture did not have any convincing effect on HRV including the HF, LF and its related components in healthy subjects. Some of the findings in the current studies are contradictory to those of Lee and colleague's study [8] (refer to Section 3.7 for details).

Since this systematic review adopted the same inclusion and exclusion criteria as Lee and colleague's study [8], the researcher suggested that the difference in findings may be due to the difference in the number of studies included. It was 12 for Lee and colleague's study [8] and 14 for the current review. This review may have included studies with higher effect sizes. Apart from this, the difference in reported risk of bias may also account for the contradictory results. The current review had a better assessment of risk of bias. For instance, in the current review, more studies were randomized (4 in Lee and colleague's study [8] while 11 in this review). Concealment is another factor. In Lee and colleague's study [8], no studies satisfied the criteria of concealment but about 75% of the selected studies in this systematic review reported concealment.

### 4.2. New Phenomena Revealed

The findings in this systematic review reveal two phenomena. First, acupuncture has reduction effect on HRV. Acupuncture decreases the LF and LF/HF ratio among nonhealthy subjects. However, the effect of acupuncture on healthy subjects is inconclusive because there were no data of nonhealthy subjects for combined analysis.

Second, acupuncture does not have any effect on the HF component of HRV. Acupuncture also has reduction effect on the HRV of sham/control. Sham/control decreases the HF for non-health subjects and HF norm for healthy subjects. Again, the effect of sham/control on healthy subjects is inconclusive because there were no data of nonhealthy subjects for combined analysis.

We find that changes in LF can change the values of LF/HF ratio or LF norm and HF norm. This is because LF or HF is a function of the LF/HF ratio or LF norm and HF norm. Therefore, it is worthwhile to look at the decreasing modulating effect of acupuncture on LF for nonhealthy subjects. LF refers to the parasympathetic influence on the balance between sympathetic and parasympathetic activities when one is in a relaxed state, like slowing heart rate, decreasing blood pressure, stimulating the gastrointestinal tract, eliminating waste, restoring energy, and building tissues. This modulating effect by acupuncture is important because, in general, parasympathetic activities represent conservative and restorative functions.

Effects of ST36 and PC6 acupoints on HRV were demonstrated that ST36 is an important acupoint of the Stomach Meridian which is documented to be related to general wellness and good *shen* (sound mind and good mood). ST36 is commonly used in acupuncture or acupressure for general health maintenance. This coincides with decrease of HF, that is, a decrease in parasympathetic activities. On the other hand, PC6 is an acupoint in the Pericardium Meridian. For nonhealthy subjects, we prefer to calm the meridian rather than exhausting it. The LF/HF is an indicator of sympathetic nerve activities; a lower number means decreased sympathetic activities or increased parasympathetic activities which may help clam the meridian. The interpretation of effect of acupoints warrants some concern because there is no information on the technique of needle insertion and no information on whether the subjects had excessive heat/cold at the time of receiving acupuncture.

### 4.3. HRV as an Indicator for the Therapeutic Effect of Acupuncture

Evidence from this systematic review partially supports the possible effect of acupuncture in modulating the low frequency component of heart rate variability. This may represent a mechanistic pathway for global physiological regulation, which is congruent with East Asian medical theory. It is important to highlight the significance of LF component of HRV. This modulation effect by acupuncture is important because, in general, parasympathetic activities represent conservative and restorative physiological activities. However, we do not have enough published work in this area to determine if HRV can be used as an indicator for the therapeutic effect of acupuncture because the dynamic change between LF and HF in maintaining the optimal status is yet to be answered in this systematic review.

### 4.4. Limitation and Further Work

We need more RCTs with high quality in this area so as to provide a direction for our evidence-based practice. We need larger sample sizes in the studies because this allows better randomization to work. The control for heterogeneity is an issue which can be overcome. We need to have studies in which both subjects and assessors can be blinded in order to minimize the psychological biases. Current studies were subject to psychological biases which would in turn affect the quality of the systematic review.

## 5. Conclusions

In this thesis, a systematic review with meta-analysis was conducted on 14 RCTs. All the studies on related HRV outcome measures showed moderate to high heterogeneity. Results showed a decreasing modulating effect of acupuncture on LF and LF/HF ratio for nonhealthy subjects and on LF norm for healthy subjects. However, the overall effect was in favour of the sham/control, not acupuncture group, for HF in nonhealthy subjects and for HF norm in healthy subjects. Evidence from this systematic review suggests that acupuncture modulates the low frequency component of HRV but not the HF component. However, we do not have enough published work in this area to determine if HRV can be an indicator for the therapeutic effect of acupuncture.

## Figures and Tables

**Table 1:** Summary of the normal values of HRV.

Component	Outcome parameter	Clinical indication
Very low frequency (VLF)	Frequency (0.0033–0.04 Hz)	Indicator of overall activity of various slow sympathetic activities
Low frequency (LF)	Frequency (0.04–0.15 Hz)	Indicator of sympathetic activities with parasympathetic influence when the respiration rate is lower than 7 per minute
High frequency (HF)	Frequency (0.15–0.4 Hz)	Indicator of parasympathetic activities
LF/HF ratio	1.5–2.0	Indicator of sympathetic activities; a higher number means increased sympathetic activities or reduced parasympathetic activities
sNormalized LF and HF (LF norm, HF norm)	Frequency (Hz)	The monotonic nonlinear transformation of LF/HF ratio. The LF/HF ratio, LF norm, and HF norm indicate the same aspects of sympathovagal balance of autonomic nervous system

**Table 2:** Inclusion criteria of the RCTs.

Component	Criteria
Participant	Adults (aged ≥18) were treated with needle acupuncture with or without electric stimulation
Intervention	Trials employed acupuncture as the sole treatment
Comparator	Trials compared needle acupuncture with any type of sham acupuncture or no treatment (control)
Outcome measures	Trials used spectral analysis of HRV as the outcome measure

**Table 3:** Summary of extracted RCTs.

Authors (year) and origin	Design sample size Conditions	Intervention (regimen)	Heart rate variability	Main results of intergroup differences	Risk of bias
Kwak et al. [22], South Korea	Parallel; 42 healthy students with examination stress	(A) AT (*n* = 14); (B) sham Tx ( relaxation therapy, *n* = 14); (C) Co (no Tx, *n* = 14)	(1) HRT (beats/min); (2) SDNN (ms); (3) SDSD (ms); (4) LF norm (n.u.); (5) HF norm (n.u.); (6) LF/HF	(1) A→, B→, C↓ when comparing between examination and normal time: *P* < 0.05; (1), (4), and (6) A↓, B→, C→ before and after examination: *P* < 0.05; (2) A↑, B→, C→ at normal time: *P* < 0.05; (5) A↑, B→, C→ before and after examination: *P* < 0.05	U, U, U, U, U

Huang et al. [23], Taiwan	Parallel; 111 healthy subjects	(A) AT (P6, 20 min on both forearms, *n* = 39); (B) sham AT ( sham acupuncture at dummy points, 20 min, *n* = 38); (C) Co: no Tx, *n* = 34)	(1) Mean RR interval (ms); (2) SD of RR interval (ms); (3) coefficient of variation (%); (4) HF norm (nu); (5) LF norm (nu); (6) LF/HF	(1) A↑, B↑, C→: *P* < 0.05; (4) A↑, B→, C→: *P* < 0.05	U, N, N, N, N

Chang et al. [24], Taiwan	Crossover, 15 healthy subjects	(A) EA (2 Hz, 30 min, *n* = 15); (B) Sham EA (minimal penetration on nonacupuncture point, no electric stimulation, *n* = 15); (C) EA plus atropine injection; sessions A, B, C in a randomized order with 3 days between sessions	(1) LF/HF; (2) LF; (3) HF	(1) A↑, B→: NS; (2), (3) A→, B→: NS	U, N, Y, U, U

Chang et al. [25], Taiwan	Crossover, 15 healthy subjects	(A) EA (2 Hz at Zusanli, 30 min, *n* = 15); (B) EA (2 Hz at Shousanli, 30 min, *n* = 15); (C) EA (100 Hz at Zusanli, 30 min, *n* = 15); (D) EA (100 Hz at Shousanli, 30 min, *n* = 15); (E) sham EA (minimal penetration on nonacupuncture point, no electric stimulation, *n* = 15); Sessions in a randomized order with 3 days between sessions	(1) LF/HF; (2) LF; (3) HF	(1) A↑ during and after EA, B→, C→, D→, E→: NS; (2) A↑ during and after EA, B→, C→, D→, E→: NS; (3) A↓ during and after EA, B→, C→, D→, E→: NS	U, N, Y, U, U

Hsu et al. [26], Taiwan	Crossover, 10 healthy subjects	(A) EA (2 Hz at BL15, 10 min, *n* = 10); (B) sham EA (*n* = 10)	(1) LF norm; (2) HF norm; (3) LF/HF; (4) HRT	(1)–(4) A↓, B→: *P* < 0.05	U, N, U, U, U

Hsu et al. [27], Taiwan	Crossover, 10 healthy subjects	(A) AT (scalp-Sishencong, 10 min, *n* = 10); (B) AT (Auricular-Shenmen, 10 min, *n* = 10); (C) sham AT (*n* = 10)	(1) LF norm; (2) HF norm; (3) LF/HF; (4) HRT	(1)–(4) A↓, B↑, C→: *P* < 0.05	U, N, U, U, U

Kim et al. [28], South Korea	Crossover, 38 female subjects with regular menstrual cycle	(A) AT (LI4 and SP6, 15 min, *n* = 38); (B) sham AT (minimal penetration on the same acupoints, 15 min, *n* = 38); One-month washout between Tx	(1) LF; (2) HF; (3) LF/HF	(1) A↑, B↑: NS; (2) A↑, B↑: *P* < 0.001 and <0.005; (3) A↓, B↓: *P* = 0.001 and NS	U, N, Y, U, U

Streitberger et al. [16], Germany	Crossover, 20 healthy subjects	(A) AT (LI4, 5 min and 15 sec manual stimulation, *n* = 20); (B) sham AT (blunted placebo needle with no penetration on the same acupoints, 5 min and 15 sec manual stimulation, *n* = 20)	(1) LF; (2) HF; (3) LF/HF	(1) and (2) A→, B→: NS; (3) A→, B→: NS, but A↑ during stimulation: NS	Y, N, Y, Y, U

Li et al. [29], China	Parallel, 29 male healthy subjects (after 3 hr driving workout)	(A) AT (15 min, *n* = 10); (B) sham AT (minimal penetration on nonacupuncture points, *n* = 10); (C) Co (with driving, 15 min, *n* = 9)	(1) LF/HF; (2) LF norm; (3) HF norm	(1)-(2) A↓, B→: *P* < 0.05; (3) A↑, B→: *P* < 0.05	U, N, Y, U, U

Chang et al. [30], Taiwan	Parallel, 12 male healthy subjects with no neurological diseases	(A) AT (PC6, 3 sessions with one-week washout, 30 min, *n* = 6); (B) sham AT (minimal penetration on nonacupuncture points, 3 sessions with one-week washout, 30 min, *n* = 6)	(1) VLF; (2) LF; (3) HF; (4) LF/HF	NS for all cases	U, N, U, U, U

Kurono et al. [31], Japan	Crossover, 14 male healthy subjects	(A) AT (epifascial stimulation at CV17 and CV16, needle inserted for 1s only, *n* = 14); (B) sham AT (AT on nonacupoints, *n* = 14)	(1) LF; (2) HF; (3) LF/HF	(1)-(2) A↑ in CV17, but not CV16; (3) NS for both CV17 and CV16	U, N, Y, U, U

Liu et al. [32], USA	1st phase (acute); Crossover, 27 patients with functional dyspepsia (Rome II)	(A) TEA (25 Hz, PC6 and ST36, 30 min, 2 sessions, *n* = 27); (B) Sham TEA (TEA was performed on sham acupoints, 30 min, 2 sessions, *n* = 27); (C) Co (electrodes were placed in the acupoints but not turned on, *n* = 27)	(1) HF; (2) LF/HF	(1) A↑ during 1st 30 min Tx, B→, C→: *P* = 0.01; (2) A↓, B→, C→: *P* < 0.05	Y, N, U, U, U

Liu et al. [32], USA	2nd phase (chronic); Crossover, 27 patients with functional dyspepsia (Rome II)	(A) TEA for 2 weeks (twice daily, 30 min), 1 week-wash-out, sham TEA for 2 weeks; (B) viceversa	(1) HF; (2) LF/HF	(1) A↑ after 2 weeks, B→, C→: *P* = 0.05; (2) A↓, B→, C→: *P* < 0.05	Y, N, Y, Y, U

Shi et al. [33], China	Crossover, 20 patients with coronary heart disease	(A) AT (30 min, *n* = 20); (B) EA (30 min, *n* = 20); (C) Co (no Tx, *n* = 20)	(1) LF; (2) HF; (3) LF/HF	(1) A↓, B↓, C→: *P* < 0.05; (2) NS for all groups; (3) A→, B↓, C→: *P* < 0.05	U, N, U, U, U

AT: acupuncture treatment; EA: electroacupuncture; TEA: transcutaneous electroacupuncture; Co: control; Tx: treatment.

Risk of bias (1) sequence generation; (2) incomplete data; (3) patient-blinded; (4) assessor blinded; (5) allocation concealment performed.

**Table 4:** A summary of the heterogeneity for random effect.

Outcomes	No. of studies			Heterogeneity Chi-sq test			Higgins *I* ^2^ test		
	All studies	Healthy	Non-healthy	All Studies	Healthy	Non-healthy	All studies	Healthy	Non-healthy
HF	9	7	2	41.31 (*P* = 0.0003)	16.99 (*P* = 0.11)	24.31 (*P* < 0.0001)	64%	35%^#^	88%
LF	7	6	1	51.93 (*P* < 0.0001)	47.44 (*P* < 0.0001)	0.15 (*P* = 0.70)	77%	79%	0%
LF/HF ratio	11	9	2	977.50 (*P* < 0.0001)	913.49 (*P* < 0.0001)	0.75 (*P* = 0.86)	98%	99%	0%
HF norm	5	5	—	32.35 (*P* < 0.0001)	32.35 (*P* < 0.0001)	—	78%	78%	NA
LF norm	5	5	—	15.39 (*P* = 0.03)	15.39 (*P* = 0.03)	—	55%	55%	NA
LI4 on HF	2	—	—	0.00 (*P* = 0.99)	—	—	0%	—	—
LI4 on LF/HF ratio	4	—	—	73.89 (*P* < 0.0001)	—	—	93%	—	—
LI4 on HF norm	2	—	—	26.62 (*P* < 0.0001)	—	—	89%	—	—
LI4 on LF norm	2	—	—	13.43 (*P* = 0.004)	—	—	78%	—	—
PC6 on HF	3	—	2	24.71 (*P* = 0.0002)	—	24.31 (*P* < 0.0001)	80%	—	0%
PC6 on LF	2	—	—	6.33 (*P* = 0.10)	—	—	53%	—	—
PC6 on LF/HF ratio	6	—	2	85.78 (*P* < 0.0001)	—	0.75 (*P* = 0.86)	88%	—	0%
PC6 on HF norm	3	—	—	28.68 (*P* < 0.0001)	—	—	86%	—	—
PC6 on LF norm	3	—	—	15.22 (*P* = 0.004)	—	—	74%	—	—
ST36 on HF	3	2	—	33.34 (*P* < 0.0001)	0.49 (*P* = 0.78)	—	88%	0%	—
ST36 on LF	2	—	—	7.39 (*P* = 0.02)	—	—	88%	—	—
ST36 on LF/HF ratio	3	—	—	108.59 (*P* < 0.0001)	—	—	95%	—	—

^#^Higgins *I* ^2^ Test is ≤50%; fixed effect is used.

**Table 5:** A summary of the effect of acupuncture in HRV outcome measures in various subject groups and by different acupoints.

Outcomes	Overall effect, *Z*			Mean difference		
	All studies	Healthy	Non-healthy	All Studies	Healthy	Non-healthy
HF	4.69 (*P* < 0.0001)*	1.39 (*P* = 0.17)^#^	5.25 (*P* < 0.0001)*	0.08 [0.05, 0.10]*	0.07 [−0.03, 0.17]^#^	0.08 [0.05, 0.10]*
LF	0.03 (*P* = 0.98)	0.72 (*P* = 0.47)	2.04 (*P* = 0.04)*	0.01 [−0.38, 0.40]	0.15 [−0.26, 0.56]	−0.98 [−1.93, −0.04]*
LF/HF ratio	2.18 (*P* = 0.03)*	1.26 (*P* = 0.21)	15.82 (*P* < 0.0001)*	−0.33 [−0.63, −0.03]*	−0.26 [−0.66, 0.14]	−0.50 [−0.56, −0.44]*
HF norm	5.00 (*P* < 0.0001)*	5.00 (*P* < 0.0001)*	—	0.51 [0.31, 0.70]*	0.51 [0.31, 0.70]*	NA
LF norm	4.03 (*P* < 0.0001)*	4.03 (*P* < 0.0001)*	—	−0.30 [−0.44, −0.15]*	−0.30 [−0.44, −0.15]*	NA
LI4 on HF	15.82 (*P* < 0.0001)*	—	—	0.21 [−0.05, 0.47]	—	—
LI4 on LF/HF ratio	0.04 (*P* = 0.97)	—	—	−0.01 [−0.29, 0.27]	—	—
LI4 on HF norm	0.24 (*P* = 0.81)	—	—	1.20 [−8.64, 11.04]	—	—
LI4 on LF norm	0.36 (*P* = 0.72)	—	—	−1.45 [9.32, 6.41]	—	—
PC6 on HF	5.47 (*P* < 0.0001)*	—	5.25 (*P* < 0.0001)*	0.07 [0.05, 0.10]*	—	0.08 [0.05, 0.10]*
PC6 on LF	0.57 (0.57)	—	—	−0.05 [−0.24, 0.13]	—	—
PC6 on LF/HF ratio	2.23 (*P* = 0.03)*	—	15.82 (*P* < 0.0001)*	−0.27 [−0.51, −0.03]*	—	−0.50 [−0.56, −0.44]*
PC6 on HF norm	0.20 (*P* = 0.84)	—	—	0.80 [−6.99, 8.58]	—	—
PC6 on LF norm	0.15 (*P* = 0.88)	—	—	−0.45 [−6.42, 5.52]	—	—
ST36 on HF	4.47 (*P* < 0.0001)*	3.00 (*P* = 0.003)*	—	0.07 [0.04, 0.11]*	−99.51 [−164.56, −34.45]*	—
ST36 on LF	0.96 (*P* = 0.34)	—	—	−136.15 [−414.89, 142.59]	—	—
ST36 on LF/HF ratio	0.61 (*P* = 0.54)	—	—	−0.09 [−0.36, 0.19]	—	—

*Denoted “significant overall effect” and “significant mean difference”, respectively.

^#^Higgins *I* ^2^ test is ≤50%; fixed effect is used.
